# Periodontal Clinicoradiographic Status and Whole Saliva Soluble Urokinase Plasminogen Activation Receptor and Tumor Necrosis Factor Alpha Levels in Type-2 Diabetic and Non-diabetic Individuals

**DOI:** 10.3290/j.ohpd.b2082019

**Published:** 2021-09-30

**Authors:** Dena Ali, Sayed Saad Qasem, Jagan Kumar Baskaradoss

**Affiliations:** a Associate Professor, Department of General Dental Practice, Kuwait University, Safat, Kuwait. Concept, methodolgy, validation, investigation, resources, wrote and reviewed the manuscript, visualisation, supervision, project administration, funding acquisition, read and approved final manucript.; b Assistant Professor, Department of Bioclinical Sciences, Kuwait University, Kuwait City, Kuwait. Methodology, validation, investigation, wrote and reviewed the manuscript, read and approved final manucript.; c Assistant Professor, Department of Developmental and Preventive Sciences, Kuwait University, Kuwait City, Kuwait. Software, validation, formal analysis, data curation, wrote and reviewed the manuscript, read and approved final manucript.

**Keywords:** periodontitis, soluble urokinase-type plasminogen activator receptor, tumor necrosis factor-alpha, type-2 diabetes mellitus, unstimulated whole saliva

## Abstract

**Purpose:**

The authors hypothesise that whole saliva soluble-urokinase-type plasminogen-activator receptor (suPAR) and tumor necrosis factor-alpha (TNF-α) levels are higher in patients with poorly-controlled than well-controlled type-2 diabetes mellitus (DM) and non-diabetic controls. The aim was to assess the periodontal clinicoradiographic status and whole-salivary suPAR and TNF-α levels in type-2 diabetic and non-diabetic individuals.

**Materials and Methods:**

Patients with and without type-2 DM were included. In all patients, hemoglobin A1c (HbA1c) levels were measured. Participants were divided into 4 groups. Group 1: patients with poorly controlled type-2 DM; group 2: patients with well-controlled type-2 DM; group 3: non-diabetic patients with periodontitis; group 4: non-diabetic patients without periodontitis. Clinicoradiographic periodontal parameters (plaque index [PI], gingival index [GI], clinical attachment loss [AL], probing depth [PD] and mesial and distal marginal bone loss [MBL]) were measured. The whole saliva total protein concentration (TPC) and suPAR as well as TNF-α levels were measured. The level of statistical significance was set at p < 0.01.

**Results:**

One hundred patients (25 patients per group) were included. Scores of PI (p < 0.01), GI (p < 0.01), clinical AL (p < 0.01), PD (p < 0.01), number of missing teeth and mesial (p < 0.01) and distal (p < 0.01) MBL were statistically significantly higher in group 1 than in groups 2–4. Scores of PI, GI, clinical AL, PD, mesial and distal MBL, and numbers of missing teeth were higher in group 3 (p < 0.01) than in groups 2 and 4. The whole saliva TPC, suPAR and TNF-α levels were statistically significantly higher among patients in group 1 (p < 0.01) than in groups 2–4.

**Conclusion:**

Patients with poorly-controlled type-2 DM presented with poorer clinicoradiographic periodontal status and increased whole saliva levels of suPAR, TNF-α and TPC compared with patients with well-controlled type-2 DM and non-diabetic individuals.

A prominent risk factor of oral inflammatory conditions, including periodontitis, is diabetes mellitus (DM),^16,24^ with type-2 DM being the most prevalent form.^13^ Chronic hyperglycemia results in increased accumulation of advanced glycation end products (AGEs) in systemic and periodontal tissues.^3,39^ When AGEs interact with their receptors (RAGE), oxidative stress occurs in human gingival fibroblasts.^23,27^ Moreover, enhanced formation of reactive oxygen species in patients with poorly-controlled DM (hemoglobin A1c [HbA1c] > 7%) increases the expression of inflammatory cytokines, such as tumor necrosis factor-alpha (TNF-α).^7,26^ The TNF-α induces a variety of cellular inflammatory and immunomodulatory effects, including activation of osteoclasts and prostaglandin synthesis.^21^ Studies^18,30^ have reported significantly higher TNF-α in the unstimulated whole saliva (UWS) of patients with DM compared with non-diabetic controls. This destructive inflammatory cytokine jeopardises periodontal tissues by augmenting marginal bone loss (MBL).^21^

The urokinase-type plasminogen activator receptor (uPAR) is a glycoprotein which is attached to cell membranes of cells such as fibroblasts, macrophages, and activated T-lymphocytes. After detachment from cell membranes, its soluble structure (suPAR) is formed.^20,34^ The suPAR is expressed in varying concentrations in biological fluids such as saliva, gingival crevicular fluid, urine, and plasma;^25,31,33^ the concentration at which it is expressed is dependent upon the severity of inflammation. High serum and salivary suPAR levels have been reported in diabetic and periodontitis patients.^9,31^ In this context, suPAR has been proposed as a potential inflammatory biomarker.^28^ Studies^8,36^ have also shown that under optimal glycemic control, diabetic patients exhibit periodontal status comparable to that of periodontally healthy individuals. According to Wang et al,^36^ optimal glycemic control reduces the expression of TNF-α in body fluids and reduces the clinical parameters of periodontal inflammation (probing depth [PD] and clinical attachment loss [AL]).

To date, salivary suPAR levels remain uninvestigated in diabetic patients. Moreover, there are no studies that have assessed salivary suPAR levels in relation to glycemic status in periodontitis patients with type-2 DM. The present study is based on the hypothesis that whole saliva suPAR and TNF-α levels are higher in patients with poorly vs well controlled type-2 DM and nondiabetic controls. With this background, the aim was to compare periodontal clinicoradiographic status and whole saliva suPAR and TNF-α levels in type-2 diabetic and non-diabetic individuals.

## Materials and Methods

### Ethics Declaration

Protocols documented in the 2013 Helsinki Declaration of experiments on humans were adopted for this study. Participating individuals were required to sign an informed consent form. Patients were informed that they could withdraw from this study at any stage, and that withdrawal or declining participation was not associated with any penalties and/or consequences. Volunteers were also invited to ask questions. The ethics committee of the Health Sciences Center, Kuwait University, approved the study protocol.

### Participants and Eligibility Criteria

Patients with and without self-reported medically diagnosed type-2 DM were invited. Individuals habitually using alcohol and nicotine-containing products such as waterpipe, cigarettes, pipe, cigarillos and electronic nicotine delivery systems (ENDS), patients with diseases including type-1 DM, prediabetes, cardiomyopathy, obesity, HIV/AIDS, and kidney and hepatic diseases were excluded. Third molars, and grossly carious dentition with embedded root remnants were considered missing. Moreover, patients having undergone surgical and/or non-surgical periodontal therapy were excluded. Furthermore, pregnant/lactating females and individuals using probiotics, bisphosphonates, antibiotics, steroids, and NSAIDS in the 3 months prior to the study were not sought.

### Hemoglobin A1c

In all patients, HbA1c levels were measured using an HbA1c measurement device (Quo-Test, EKF Diagnostics; Magdeburg, Germany). The HbA1c levels were measured prior to clinicoradiographic examination by one investigator (Kappa score 0.82).

### Study Groups

Participants were classified in to 4 groups: group 1: patients with poorly controlled type-2 DM (HbA1c > 7%); group 2: patients with well-controlled type-2 DM (HbA1c ≤ 7%); group 3: non-diabetic patients with periodontitis (HbA1c < 5%); group 4: non-diabetic patients without periodontitis (HbA1c < 5%).^26^

### Questionnaire

Demographic data pertaining to family history, DM, age, educational status (university-level education), gender, most recent visit to a dental hygienist/dentist/oral healthcare provider, and daily toothbrushing and flossing were gathered using a questionnaire. Individuals who reported to have graduated from a university were categorised as having university-level education.

### Clinicoradiographic Investigations

In all groups, full-mouth plaque and gingival indices (PI and GI, respectively),^19^ clinical AL,^1^ and PD^2^ and were measured by one investigator (Kappa 0.86). Missing teeth were counted. Full-mouth digital intra-oral radiographs (Planmeca Romexis Intraoral X-Ray, Planmeca; Helsinki, Finland) were taken, and standardisation of all radiographs was done as described elsewhere.^14,35^ All radiographs were examined by one researcher (Kappa score 0.83).

### Collection of Unstimulated Whole Saliva (UWS) and Assessment of Whole Saliva TPC and suPAR, TNF-**α** Levels

In all groups, UWS samples were collected 48 h after clinicoradiographic examinations. All UWS samples were collected as described elsewhere.^37^ In summary, all UWS samples were collected during the morning (between 8:00 am and 9:00 am) with the patients being in a fasting state. Patients were comfortably seated on a chair in a quiet room and requested to allow UWS to accumulate in the oral cavity for 5 min. During this time, patients were advised to refrain from swallowing and jaw/lip movements. Patients were then requested to expectorate the UWS into a disposable plastic funnel, which was coupled to a graduated cylinder. The amount of expectorated UWS was recorded in milliliters. For each individual per group, unstimulated whole saliva flow rate (UWSFR) was determined in ml per minute (ml/min). The UWS samples were transferred to sterile plastic tubes with lids and centrifuged in a cold room at 3000 rpm for 5 min. The supernatant was collected and stored at -80°C. All UWS samples were assessed for suPAR and TNF-α levels within 24 h of collection. Levels of whole saliva suPAR (Human suPAR ELISA kit, MBS7606253; Calabasas, CA, USA), and TNF-α (SunRed Bio; Shanghai, China) were determined using enzyme-linked immunosorbent assays according to the manufacturers’ guidelines. Solutions of standard and samples were added into the wells which were precoated with TNF-α and SUPAR antibodies. To each well, streptavidin-conjugated HRP enzyme and biotin conjugated antibody were added. The plates were incubated for 60 min at 37°C, after which the wells were washed five times with 350 μl of a wash buffer. Chromogen was added and the plates were again incubated at 37°C in a dark room. The reaction was completed using a H_2_SO_4_ stop solution. An ELISA reader (ELX 800, BioTek; Winooski, VT, USA) was used at 450 nm to read the absorbances of all biomarkers, and concentrations of samples were calculated based on standard absorbance values. The whole saliva total protein concentration (TPC) was determined using the bicinchoninic acid (BCATM) Protein Assay Reagent Kit (Product No. 23227, Pierce Chemical; Rockford, IL, USA). Using albumin as the standard, aliquots of saliva (200 µl/well) were placed in microtiter plates. The protein assay reagent was added, and the plates were incubated at 37°C for 30 min. Optical densities were read at 550 nm in a microtiter plate spectrophotometer (Molecular devices, Vmax; Sunnyvale, CA, USA).

### Statistical and Power Analysis

Statistical software (SPSS, v 20; Chicago, IL, USA) was used to perform group comparisons in relation to clinicoradiographic parameters, UWSFR and whole saliva suPAR and TNF-α levels. The Kolmogrov-Smirnov test was used to assess data normality. Groupwise statistical evaluations were done using one-way ANOVA and Bonferroni post-hoc adjustment. A p-value < 0.01 was designated as an indicator of statistical significance. Sample size was estimated beforehand using data obtained from a pilot investigation (nQuery Advisor 5, Statistical Solutions; Saugus, MA, USA). Sample size estimation was based on the presumption that a mean difference of 1 mm in clinical AL and PD should be detected at a significance level of 0.01 and a desired study power of at least 80%. It was estimated that a sample size of at least 24 patients/group would achieve 95% study power with a 0.01 two-sided significance level.

## Results

### General Characteristics of the Study Participants

In total, 100 patients were included. There were 25 patients per group (group 1: 20 males and 5 females; group 2: 22 males and 3 females; group 3: 20 males and 5 females; group 4: 21 males and 4 females). There was no statistically significant difference in the mean age of participants in all groups. In groups 1 and 2, the duration of type-2 DM was 8.5 ± 1.2 and 8.8 ± 0.8 years, respectively. The mean HbA1c levels were statistically significantly higher among patients in group 1 (p < 0.01) compared with groups 2, 3 and 4. There was no statistically significant difference in the mean HbA1c levels among patients in groups 2, 3 and 4. A family history of DM was more often reported by patients in group 1 (80%) compared with individuals in groups 2 (32%), 3 (28%) and 4 (24%). University-level education was more often attained by patients in groups 2 (60%), and 4 (68%) compared with patients in groups 1 (12%) and 3 (28%). In groups 2 and 4, participants had visited a dental hygienist/dentist 9.1 ± 0.4 and 7.2 ± 0.3 months ago, respectively, compared with individuals in groups 1 (17.3 ± 3.8 months) and 3 (14.6 ± 1.7 months) (p < 0.01) (Table 1).

**Table 1:** General characteristics of the study groups

Parameters	Group 1	Group 2	Group 3	Group 4
Patients (n)	25	25	25	25
Mean age in years	50.6 ± 5.2	52.4 ± 3.7	53.1 ± 2.1	51.7 ± 2.8
Males:females	20:5	22:3	20:5	21:4
Duration of type-2 DM	8.5 ± 1.2	8.8 ± 0.8 years	NA	NA
Mean HbA1c	10.6 ± 0.3*	5.2 ± 0.07	4.8 ± 0.2	4.5 ± 0.1
Family history of DM	20 (80%)	8 (32%)	7 (28%)	6 (24%)
Recent visit to dental hygienist/dentist (in months)	17.3 ± 3.8*	9.1 ± 0.4	14.6 ± 1.7	7.2 ± 0.3
University-level education	3 (12%)	15 (60%)	7 (28%)	17 (68%)

*Compared with groups 2 (p < 0.01), 3 (p < 0.01) and 4 (p < 0.01). Group 1: patients with poorly-controlled type-2 DM; group 2: patients with well-controlled type-2 DM; group 3: nondiabetic patients with periodontitis; group 4: nondiabetic patients without periodontitis; DM: diabetes mellitus; HbA1c: hemoglobin A1c; NA: not applicable.

### Clinicoradiographic Parameters

Scores of PI (p < 0.01), GI (p < 0.01), clinical AL (p < 0.01), PD (p < 0.01) and mesial (p < 0.01) and distal (p < 0.01) MBL were statistically significantly higher among patients in group 1 compared with individuals in groups 2, 3 and 4. The numbers of missing teeth were significantly higher among patients in group 1 (p < 0.001) compared with individuals in groups 2, 3 and 4. There was no statistically significant difference in the scores of PI, GI, clinical AL, PD, mesial and distal MBL and numbers of missing teeth among patients in groups 2 and 4. Scores of PI, GI, clinical AL, PD, mesial and distal MBL and numbers of missing teeth were statistically significantly higher among patients in group 3 (p < 0.01) compared with individuals in groups 2 and 4 (Table 2).

**Table 2:** Clinicoradiographic periodontal status of the study groups

Parameters	Group 1 (n = 25)	Group 2 (n = 25)	Group 3 (n = 25)	Group 4 (n = 25)
Plaque index	3.6 ± 0.9*	0.8 ± 0.08	2.2 ± 0.06†	0.4 ± 0.005
Gingival index	3.3 ± 0.5*	1.05 ± 0.04	2.4 ± 0.03†	0.3 ± 0.02
Clinical attachment loss	6.5 ± 0.3 mm*	1.01 ± 0.2 mm	4.2 ± 0.08 mm†	0.2 ± 0.003 mm
Probing depth	5.6 ± 0.2 mm*	1.4 ± 0.08 mm	4.1 ± 0.06 mm†	0.8 ± 0.005 mm
Marginal bone loss (mesial surface)	7.2 ± 0.2 mm*	2.2 ± 0.05 mm	4.4 ± 0.05 mm†	2.06 ± 0.03 mm
Marginal bone loss (distal surface)	7.05 ± 0.2 mm*	2.04 ± 0.08 mm	4.3 ± 0.007 mm†	1.9 ± 0.005 mm
Number of missing teeth	13.5 ± 1.2 teeth*	3.4 ± 0.8 teeth	6.02 ± 0.5 teeth†	2.2 ± 0.2 teeth

*Compared with groups 2 (p < 0.01), 3 (p < 0.01) and 4 (p < 0.01). †Compared with groups 2 (p < 0.01) and 4 (p < 0.01). Group 1: Patients with poorly-controlled type-2 DM; group 2: patients with well-controlled type-2 DM; group 3: non-diabetic patients with periodontitis; group 4: non-diabetic patients without periodontitis; DM: diabetes mellitus; HbA1c: hemoglobin A1c.

### UWSFR and Whole Saliva suPAR and TNF-**α** Levels

The UWSFR was significantly lower in group 1 (p < 0.01) than in the other groups. The whole saliva TPC, suPAR and TNF-α levels were higher in group 1 (p < 0.01) than in groups 2, 3 and 4. There was no statistically significant difference in the UWSR, whole saliva TPC, and suPAR and TNF-α levels among patients in groups 2 and 4. Whole saliva TPC, and suPAR and TNF-α levels were statistically significantly higher in group 3 (p < 0.01) than in groups 2 and 4 (Table 3).

**Table 3:** Unstimulated-whole-salivary-flow-rate and whole saliva suPAR and TNF-α levels

Parameters	Group 1 (n = 25)	Group 2 (n = 25)	Group 3 (n = 25)	Group 4 (n = 25)
UWSFR (ml/min)	0.09 ± 0.05 ml/min*	0.37 ± 0.03 ml/min	0.38 ± 0.04 mm	0.41 ± 0.005 ml/min
Whole saliva total protein concentration (μg/ml)	2084.7 ± 87.6 μg/ml*	994.2 ± 64.9 μg/ml	1127.2 ± 69.4 μg/ml†	806.4 ± 79.4 μg/ml
suPAR (ng/μl)	19.06 ± 2.74 ng/μl	2.42 ± 0.29 ng/μl	8.9 ± 1.3 ng/μl†	1.71 ± 1.25 ng/μl
TNF-α (pg/ml)	15.24 ± 3.2 pg/ml	3.51 ± 0.68 pg/ml	7.1 ± 0.5 pg/ml†	2.82 ± 0.14 pg/ml

*Compared with groups 2 (p < 0.01), 3 (p < 0.01) and 4 (p < 0.01). †Compared with groups 2 (p < 0.01), and 4 (p < 0.01). UWSFR: unstimulated whole saliva flow rate; suPAR: soluble urokinase plasminogen activating factor; TNF-α: tumor necrosis factor-alpha.

### Correlation of HbA1c Levels with Clinical AL and PD

**Fig 1:**
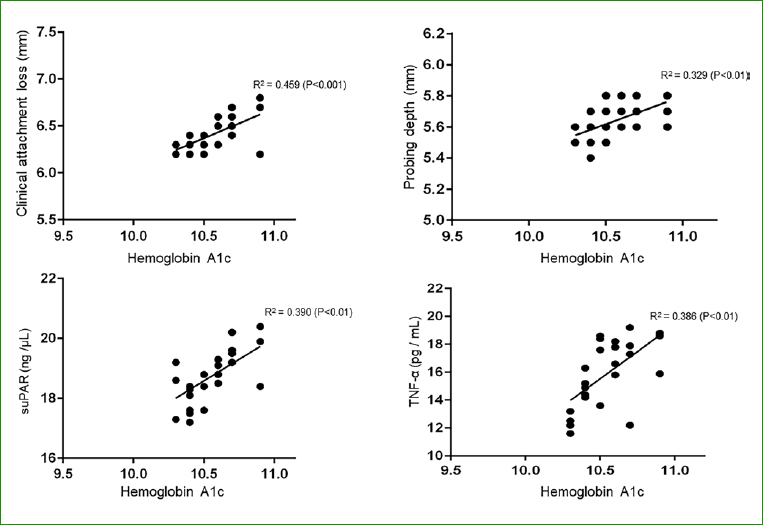
Correlation of HbA1c levels with clinical AL and PD in group 1.

In group 1, a statistically significant correlation was observed for HbA1c levels and clinical AL (p < 0.001) and PD (p < 0.01) (Fig 1). In groups 2 and 4, there was no statistically significant correlation between HbA1c levels and clinical AL and PD (data not shown).

### Correlation of HbA1c Levels with Whole Saliva suPAR and TNF-**α** Levels as well as Total Protein Concentration (TPC)

**Fig 2:**
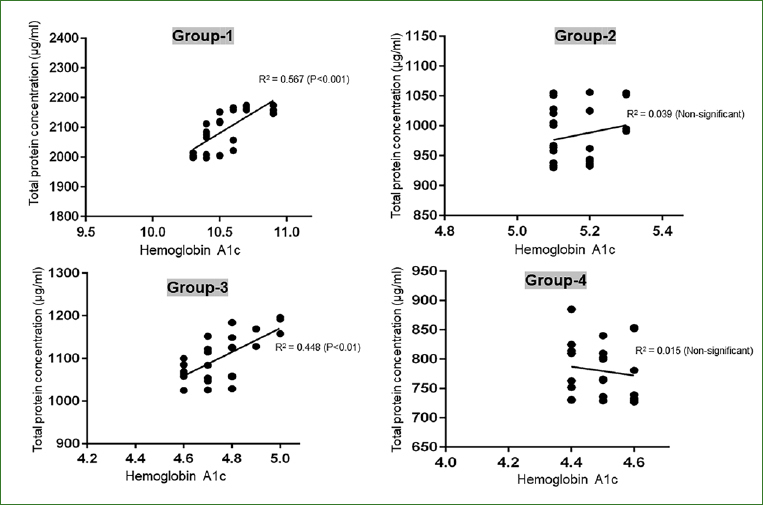
Correlation of HbA1c levels with whole saliva suPAR and TNF-α levels as well as total protein concentration in all groups.

In group 1, a statistically significant correlation was observed between increasing HbA1c levels and whole saliva suPAR (p < 0.01) and TNF-α levels (p < 0.01) (Fig 1). The whole saliva TPC was statistically significantly corelated with HbA1c levels in groups 1 and 3 (Fig 2). In groups 2 and 4, there was no statistically significant correlation between HbA1c and whole saliva TPC, suPAR and TNF-α levels (data not shown).

## Discussion

From a clinical and radiographic point of view, our results demonstrated that the clinicoradiographic parameters of periodontal inflammation were worse in patients with poorly-controlled than well-controlled type-2 DM and non-diabetic controls. These results are in accordance with previous clinical studies.^4,6,15^ However, the present study is the first to assess whole saliva suPAR levels in relation to glycemic control among type-2 diabetic patients. The present results clearly demonstrated that levels of suPAR and TNF-α were significantly higher in the UWS samples collected from patients with poorly-controlled compared to individuals with well-controlled type-2 DM. Moreover, the whole saliva TPC level was also statistically significantly higher among patients with poorly-controlled type-2 DM in contrast to other groups. It is challenging to determine the precise mechanism that may have raised the level of whole saliva TPC and expressions and concentrations of suPAR and TNF-α among patients in group 1 (patients with poorly-controlled type-2 DM) compared with other groups. One factor that may have contributed in this scenario is the state of persistent hyperglycemia among patients in group 1. Chronic hyperglycemia is known to enhance the production and accumulation of AGEs in the periodontal tissues,^12^ which in turn induces a state of oxidative stress in the targeted tissues, thereby augmenting inflammation.^12^ In addition, according to Sonnenschein and Meyle,^32^ persistent hyperglycemia alters the function of leukocytes and produces reactive oxygen species than enhance the expression and concentrations of destructive inflammatory cytokines (such as TNF-α, IL-1-beta and IL-6) in body fluids, including UWS. A critical evaluation of pertinent indexed literature revealed no studies that had correlated whole saliva suPAR levels with expression of AGEs among patients with type-2 DM. However, an exhaustive review of indexed databases found one study^38^ in which the authors assessed serum suPAR levels in relation to RAGE in patients with acute respiratory distress syndrome (ARDS). The results showed that serum suPAR and RAGE levels were correlated with the severity of ARDS.^38^ Given the results reported by Yang et al,^38^ it is theoretically possible that whole saliva suPAR and TNF-α are correlated with an increased expression of AGEs in the UWS of patients with poorly-controlled compared vs well-controlled type-2 DM and non-diabetic controls. These results suggest that whole saliva suPAR may be a potential biomarker of periodontal inflammation. Nevertheless, and further well-designed and power-adjusted studies are warranted to validate this hypothesis.

An interesting finding in the present study was that linear regression analysis showed that clinical AL and PD were the only clinical parameters that correlated with HbA1c levels among patients with poorly-controlled type-2 DM. Moreover, whole saliva TPC and suPAR levels also demonstrated a statistically significant increase as HbA1c levels among patients in group 1. One explanation for this can be associated with an experimental study^10^ on Akita mice, in which experimentally-induced hyperglycemia was shown to predispose the rodents to an exaggerated inflammatory response and leukocyte dysfunction. Similarly, in another experimental study on mice, Zhang et al^40^ assessed the molecular mechanisms by which hyperglycemia drives inflammation-induced aging in the gingival tissue of diabetic mice. The results showed that hyperglycemia increases senescence of macrophage-infiltrated and gingival tissues.^40^ However, under optimal glycemic control, it is speculated that aging of gingival tissues is markedly less, as reflected by significantly lower probing depths, clinical AL and whole saliva TPC and suPAR and TNF-α levels. In this context, it is mandatory to educate the public, especially and including patients with DM, about the detrimental effects of hyperglycemia and beneficial effects of glycemic control on dental and overall health. Routine community-driven patient education programs and efforts may play a role in this regard. In addition, oral health education and promotion in the community is also recommended by the authors.

One limitation of the present investigation is that the study design was observational. In other words, periodontitis was not treated. Mechanical debridement of periodontal tissues and tooth surfaces (scaling and root planing [SRP]) is the classical treatment strategy for periodontal diseases, including periodontitis.^5^ However, adjunct therapies, e.g. essential-oil-based oral rinses and photodynamic therapy (PDT), have been reported to be more effective in the treatment of periodontitis compared with SRP alone.^11^ Moreover, it has also been proposed that SRP helps improve metabolic control in patients with DM.^29^ The authors of the present study speculate that SRP, when performed with adjunct therapies such as those mentioned above, is more effective in reducing periodontal inflammation and HbA1c levels in patients with type-2 DM, and furthermore that such an interventional strategy would reduce the expression and concentrations of inflammatory biomarkers including suPAR and TNF-α in the UWS of diabetic patients. It is well established that tobacco smoking is a risk factor for periodontitis,^17,22^ and that habitual smoking compromises the outcomes of oral therapeutic interventions.^22^ It is therefore hypothesised that the outcomes of SRP (regardless of the use of adjuvant interventions) are compromised and whole saliva suPAR and TNF-α levels remain unchanged in habitual tobacco smokers. Further studies are needed to test the aforementioned hypotheses.

## Conclusions

Patients with poorly-controlled type-2 DM present with poorer clinicoradiographic periodontal status and increased whole saliva levels of suPAR, TNF-α and TPC compared with patients with well-controlled type-2 DM and non-diabetic individuals.
